# Scurvy Still Exists: A Case of Severe Vitamin C Deficiency With Functional Decline in the United States

**DOI:** 10.7759/cureus.99377

**Published:** 2025-12-16

**Authors:** Alberto Nenninger Leon, Chandan S Sapkota

**Affiliations:** 1 Internal Medicine, San Juan Regional Medical Center, Farmington, USA; 2 Internal Medicine, Weiss Memorial Hospital, Chicago, USA

**Keywords:** corkscrew hair, diagnostic delay, empiric treatment, functional decline, gingival bleeding, malnutrition, nutritional assessment, perifollicular hemorrhage, scurvy, vitamin c deficiency

## Abstract

Scurvy remains a real, albeit underdiagnosed, condition in developed nations. Although most Western countries have introduced vitamin-fortified foods across nearly all supermarket categories, cases continue to occur and are frequently overlooked. Patients often undergo extensive and costly laboratory and imaging evaluations before the simple yet elusive diagnosis is established.

No formal U.S. analyses have quantified the total healthcare expenditures attributable to delayed diagnosis of scurvy. However, data from the Malnutrition Quality Improvement Initiative indicate that hospitals treating malnourished patients incur an average cost of USD 25,500 per admission, compared with approximately USD 12,500 for standard inpatient care.

We report the case of a 50-year-old woman presenting with profound weakness, anemia, and functional decline. Despite extensive prior evaluation, the diagnosis was initially missed. Careful physical examination and dietary assessment revealed clinical signs of vitamin C deficiency, subsequently confirmed by undetectable serum ascorbic acid levels. The patient improved rapidly following vitamin C supplementation. This case highlights the enduring importance of nutritional assessment and bedside examination in recognizing preventable diseases.

## Introduction

Vitamin C deficiency, or scurvy, is uncommon in modern clinical practice, particularly within developed economies, yet it continues to affect vulnerable populations. Historically, scurvy was a major public health problem and a significant barrier to global exploration and commerce. Prior to the Industrial Revolution, and especially during the Victorian era, it caused widespread morbidity and mortality among sailors on long voyages, severely limiting naval operations.

Clinically, affected individuals developed bleeding gums, poor wound healing, fragile joints, and profound fatigue, symptoms that often incapacitated crews and undermined maritime expeditions. It is estimated that scurvy accounted for up to 50% of deaths on long-distance voyages, far exceeding losses due to storms or conflict. The discovery that scurvy resulted from vitamin C deficiency, and its subsequent prevention through citrus supplementation, marked a pivotal advancement in naval medicine and contributed substantially to the operational success of the British Empire.

Although now rare, scurvy persists in individuals with food insecurity, psychiatric illness, or social isolation [1,2]. Its nonspecific presentation often leads to delayed diagnosis in modern settings. This case highlights the clinical implications of missed early recognition and reinforces the continuing importance of a thorough physical examination in identifying nutritional deficiencies [3].

## Case presentation

We present the case of a 45-year-old woman who arrived at a rural hospital in the southwestern United States following a fall and progressive weakness. Her initial complaints were nonspecific, consisting mainly of fatigue, malaise, and increasing difficulty performing basic activities of daily living. She had become largely dependent on others for sustenance and self-care. On initial assessment, her presentation appeared consistent with functional decline of unclear etiology. The admitting team requested hospitalization primarily based on a physical therapy evaluation, anticipating that she would likely require skilled nursing placement upon discharge.

However, beneath this seemingly straightforward presentation emerged a far more complex history. The patient’s symptoms were vague and inconsistent with any clear diagnostic category, prompting further exploration of her social circumstances. Through careful questioning, it was revealed that she had recently relocated due to personal and financial hardships and was now residing with relatives. Her diet had consisted almost entirely of crackers and water for several months, resulting in significant unintentional weight loss and clinical malnutrition. These findings, initially overlooked, reframed the diagnostic approach considerably.

Further discussion revealed that her decline had evolved insidiously over nearly six months. Following a divorce, she had experienced a period of depression characterized by social withdrawal, prolonged bed rest, and minimal physical activity. Although she sought medical attention early in her illness, her concerns were met with nonspecific diagnoses, and she was prescribed an SSRI that she never initiated due to a clerical error with the pharmacy. Over time, she developed increasing distrust toward medical professionals after a series of extensive yet inconclusive evaluations. Records from a tertiary care center documented consultations with approximately seven specialists, multiple MRI scans of the brain and spine, two electromyography studies, and comprehensive laboratory testing, including negative rheumatologic panels such as antinuclear antibody (ANA), antineutrophil cytoplasmic antibody (ANCA), and rheumatoid factor (RF), among others, all without diagnostic clarification. These evaluations also contributed to worsening financial strain, ultimately forcing her to relocate closer to family as she became unable to care for herself independently.

Upon arrival at our institution, the patient was tangential in conversation and a poor historian, often digressing or minimizing her symptoms. Collateral information from a close family member proved essential in reconstructing the trajectory of her illness. It became evident that she had spent recent months largely confined to a small, dimly lit room with minimal nourishment and almost no sunlight exposure. Please refer to Table 1 for correlation with laboratory findings.

**Table 1:** Initial Laboratory Results

Parameter	Result	Reference Range	
Hemoglobin	6 g/dL	12–16 g/dL (F); 13.5–17.5 g/dL (M)	
White blood cell count (WBC)	15 ×10⁹/L	4–10 ×10⁹/L	
Platelets	434 ×10⁹/L	150–400 ×10⁹/L	
Red cell distribution width (RDW)	17%	11.5–14.5%	
Reticulocyte index	4.3%	0.5–2.0%	
Sodium	132 mmol/L	135–145 mmol/L	
Bicarbonate (HCO₃⁻)	15 mmol/L	22–28 mmol/L	
Creatinine	0.5 mg/dL	0.6–1.3 mg/dL	
Total bilirubin	2.7 mg/dL	0.2–1.2 mg/dL	
Direct bilirubin	1.0 mg/dL	0.0–0.3 mg/dL	
Ferritin	32 ng/mL	30–400 ng/mL (M); 15–150 ng/mL (F)	
Total iron-binding capacity (TIBC)	301 μg/dL	250–450 μg/dL	
Serum iron	62 μg/dL	60–170 μg/dL	
Transferrin saturation	21%	20–50%	
Lactate dehydrogenase (LDH)	135 U/L	140–280 U/L	
Thyroid-stimulating hormone (TSH)	5.5 mIU/L	0.4–4.5 mIU/L	
International normalized ratio (INR)	1.5	0.8–1.2	

Physical examination findings

On examination, the patient was noted to have poor dentition with diffuse gingival bleeding. Cutaneous findings included perifollicular petechiae, corkscrew hairs, and multiple ecchymoses with a maculopapular rash predominantly involving the lower extremities. Muscle strength was reduced (4/5) in all extremities, with diminished patellar reflexes bilaterally.

Given her characteristic dermatologic findings and clear evidence of malnutrition, empiric vitamin C supplementation was promptly initiated. A comprehensive evaluation ruled out major comorbidities. Repeat spinal imaging revealed no acute or structural abnormalities, and gastrointestinal studies were negative for occult bleeding. A hematologic panel was also obtained and was negative for evidence of autoimmune hemolysis. The patient was started on a structured physical therapy program, which she initially met with reluctance but gradually accepted as her hospitalization progressed.

Subsequent confirmatory testing revealed an undetectable serum vitamin C concentration (<0.1 mg/dL), establishing the diagnosis of scurvy. Notably, her condition improved within days of supplementation, with rapid resolution of fatigue and progressive recovery of strength and mood. By the midpoint of her hospitalization, she was also started on a daily multivitamin with iron supplementation. She was ultimately discharged to a skilled nursing facility for continued rehabilitation, demonstrating marked physical and psychological improvement at the time of transfer.

## Discussion

Scurvy, once thought to be eradicated in developed countries, still persists among at-risk populations [4]. According to NHANES data, about 7.1% of U.S. adults are vitamin C deficient, with disproportionately higher rates among low-income individuals and smokers [5].

Vitamin C is essential for collagen synthesis, iron absorption, and neurotransmitter production [6]. Clinical deficiency may manifest as gingival disease, petechiae, ecchymoses, fatigue, irritability, and depression [7]. These symptoms are nonspecific and are often misattributed to other chronic conditions.

In this case, despite months of intensive medical evaluation, scurvy was not considered. This reflects a broader diagnostic gap in current practice, namely a lack of attention to nutritional history and physical signs. Notably, in 85% of adult U.S. cases of scurvy, diagnosis is delayed by more than six weeks [6].

Treatment, however, is simple and effective. Vitamin C dosages of 100-500 mg daily typically resolve symptoms within 48-72 hours [6]. Our patient experienced this rapid improvement following oral supplementation and supportive care.

## Conclusions

Scurvy remains a relevant diagnosis in modern clinical practice, particularly among individuals with risk factors such as poor dietary intake, food insecurity, psychiatric illness, or social isolation. Although often considered a disease of the past, its reemergence in vulnerable populations highlights persistent gaps in nutritional access and health literacy. Historically, the recognition and systematic prevention of scurvy represented one of the earliest triumphs of evidence-based public health. The British Royal Navy’s adoption of citrus juice in the late 18th century led to the near elimination of the disease among sailors. By 1780, approximately 1,500 cases were recorded in Portsmouth, whereas by 1800, fewer than ten remained, a striking demonstration of the power of preventive nutrition.

This case underscores the enduring importance of maintaining a high index of suspicion for nutritional deficiencies, even in developed healthcare settings. Bedside diagnostic clues, such as gingival bleeding, perifollicular petechiae, and corkscrew hairs, remain invaluable, particularly when laboratory findings or imaging prove nonspecific. Early recognition and prompt vitamin C supplementation can lead to rapid clinical improvement, prevent significant morbidity, and reduce unnecessary diagnostic testing or prolonged hospitalization. In this instance, timely diagnosis and treatment not only reversed the patient’s physical decline but also restored her confidence and independence. Within weeks, she regained functional strength, resumed social engagement, and began rebuilding her personal and professional life, an outcome that reflects both the resilience of the human body and the lasting value of attentive, comprehensive clinical assessment.
